# Recent trends in lipid metabolism research in liver cancer: a bibliometric analysis

**DOI:** 10.3389/fonc.2026.1704007

**Published:** 2026-03-19

**Authors:** Yaru Shi, Xinyu Yang, Yunfeng Yu, Yanan Bai, Pei Liu, Jianzhong Cao, Weibin Xie

**Affiliations:** 1 Department of Integrated Traditional Chinese and Western Medicine, Hunan Cancer Hospital and The Affiliated Cancer Hospital of Xiangya School of Medicine, Central South University, Changsha, China; 2 School of Traditional Chinese Medicine, Hunan University of Chinese Medicine, Changsha, Hunan, China

**Keywords:** bibliometrics, Citespace, lipid metabolism, liver cancer, VOSviewer

## Abstract

**Introduction:**

This study aims to systematically map the intellectual landscape and emerging trends in lipid metabolism research within hepatocellular carcinoma from 2014 to 2024.

**Methods:**

A total of 607 peer-reviewed publications were retrieved from the Web of Science Core Collection and PubMed. Bibliometric and visualization tools, including VOSviewer and CiteSpace, were employed to perform data analysis, including keyword co-occurrence and cluster analysis.

**Results:**

We identified a significant surge in research activity, with 53.05% of the total literature published in the last three years. China and the USA emerged as the leading contributors, with the University of California System and the journal Cancers being the most prolific institution and publication outlet, respectively. Current research hotspots are centered on the mechanisms by which oxidative stress drives the transformation of non-alcoholic fatty liver disease into hepatocellular carcinoma. Furthermore, three critical frontiers for future investigation were identified: (1) the regulatory role of PPARγ in lipid metabolic reprogramming and its therapeutic implications; (2) the molecular mechanisms of the farnesoid X receptor in modulating bile acid metabolism during hepatocarcinogenesis; and (3) the NF-κB signaling pathways that mediate metabolic shifts and confer chemoresistance in liver cancer.

**Discussion:**

These findings provide a comprehensive reference for prioritizing research directions and therapeutic target discovery in the metabolic-related oncology domain.

## Introduction

According to global cancer registration data, as of 2022, liver cancer ranks sixth in terms of incidence and is the third leading cause of cancer-related deaths (1). It is projected that by 2025, over one million individuals will be diagnosed with liver cancer annually. Primary liver cancer is classified into three major types: hepatocellular carcinoma (HCC), intrahepatic cholangiocarcinoma, and angiosarcoma, with HCC being the most prevalent and accounting for 75% to 85% of all cases (2). The pathogenic factors include various viruses, liver cirrhosis, fibrosis, smoking, obesity, and diabetes (3). Common treatment modalities encompass hepatectomy, liver transplantation, radiotherapy, chemotherapy, immunotherapy, ablation, targeted therapy, and nanotechnology (4). Surgical resection remains the only treatment option that may potentially cure liver cancer. However, due to the absence of specific clinical symptoms in the early stages of liver cancer, most patients are diagnosed at intermediate or advanced stages, thereby missing the optimal surgical window (5). This clinical dilemma results in very limited treatment options and poor prognoses for patients with advanced liver cancer. Therefore, enhancing early screening, improving diagnostic capabilities, and developing novel and effective treatment strategies have become critical issues that urgently need to be addressed in the current liver cancer research landscape (6).

Lipid metabolism plays a crucial regulatory role in the occurrence and development of tumors, involving two key metabolic pathways: fatty acid synthesis and the mevalonate pathway (7, 8). Relevant studies have demonstrated that abnormal lipid metabolism and the resulting lipid metabolic reprogramming are significant driving factors in tumor occurrence and progression (9). The intrinsic mechanisms primarily include the aberrantly activated lipid synthesis pathway, such as the overexpression of fatty acid synthase regulated by sterol regulatory element-binding protein 1, which provides membrane structures and energy sources for tumor cells; genomic instability resulting from the accumulation of lipid peroxides under oxidative stress; and immune escape facilitated by lipid metabolic reprogramming within the tumor microenvironment. Particularly in liver cancer, the increase in monounsaturated fatty acid synthesis mediated by stearoyl-CoA desaturase significantly promotes tumor cell proliferation and metastasis (10, 11). A comprehensive understanding of the relationship between abnormal lipid metabolism and the occurrence of liver cancer is vital for improving diagnosis and treatment strategies.

In recent years, bibliometric analyses in oncology have predominantly focused on the tumor immune microenvironment (TIME) and interventional therapies (12, 13), or explored psychosocial dimensions such as quality of life and social isolation (14, 15). While these studies have provided valuable insights into tumor progression, interventions, and patient care, there remains a conspicuous paucity of bibliometric evaluations specifically interrogating the metabolic drivers of hepatocarcinogenesis (16). With the global etiological shift of liver cancer from viral hepatitis to metabolic dysfunction-associated steatotic liver disease (MASLD), lipid metabolic reprogramming has emerged as a core mechanism equally critical to immune evasion (17). Distinct from previous studies that treat metabolism as a peripheral topic, our analysis is exclusively dedicated to the landscape of lipid metabolism. Our primary objective is not merely to map publication trends, but to critically interrogate the translational gap between robust basic mechanistic discoveries and the current stagnation in clinical targeted therapies. By juxtaposing these bibliometric hotspots with clinical realities, this study provides a perspective that is fundamentally distinct from existing immunological or epidemiological bibliometric reviews.

## Materials and methods

### Data sources and methods

We used PubMed and Web of Science Core Collection (WoSCC) as the data source for literature retrieval. The specific search query formula is as follows: TS = (“Hepatic Neoplasms” OR “Hepatic Neoplasm” OR “Neoplasm, Hepatic” OR “Neoplasms, Hepatic” OR “Neoplasms, Liver” OR “Liver Neoplasm” OR “Neoplasm, Liver” OR “Cancer of Liver” OR “Liver Cancer” OR “Cancer, Liver” OR “Cancers, Liver” OR “Liver Cancers” OR “Hepatocellular Cancer” OR “Cancers, Hepatocellular” OR “Hepatocellular Cancers” OR “Cancer of the Liver” OR “Cancer, Hepatocellular” OR “Hepatic Cancer” OR “Cancer, Hepatic” OR “Cancers, Hepatic” OR “Hepatic Cancers”) AND TS = (Lipid metabolism). To ensure data completeness and reproducibility, a comprehensive literature search was conducted on July 6, 2025, covering the period from January 1, 2014, to December 31, 2024. Only articles and reviews published within this predefined window were included in the subsequent bibliometric analysis. Inclusion criteria (1): Only articles and reviews are included; (2) The literature must be in English. Exclusion criteria (1): Retracted literature; (2) Literature irrelevant to the research content.

### Analytical tools and methods

The data were imported into Microsoft Excel 2021 to build a bibliometric database. VOSviewer 1.6.20 (18), Bibliometrix 4.3.1 (19), Scimago Graphica 1.0.4 (20), and CiteSpace 6.2.4 (21) were used to conduct bibliometric analysis on the research data, including countries, institutions, journals, authors, citations, and keywords. The study quantitatively analyzed multiple bibliometric indicators, including average citation per item (ACI), H-index, and betweenness centrality (22, 23). The ACI indicator is used to measure the academic influence of researchers, organizations, or academic journals, with a higher value indicating greater academic influence (24). The H-index refers to the highest number of papers that have received at least one citation. The higher the H-index, the greater the influence. Betweenness centrality is a crucial indicator utilized to identify the relative importance of nodes within a network. It measures a node’s capacity to act as a bridge along the shortest paths between other nodes. In this study, nodes with a betweenness centrality score exceeding 0.1 are considered significant hub nodes in the collaboration networks (25).

VOSviewer was used for the analysis of author collaboration networks, and institutional association maps. To ensure visual clarity in VOSviewer, the minimum occurrences for institutions and authors were set to 4 and 2, respectively. Each node in VOSviewer represents an entity, with its size being proportional to its weight. The thickness of the connections between nodes reflects the strength of collaboration, co-citation, or co-occurrence. CiteSpace was used for visualization analysis of journal dual maps, research topic timelines, keyword clustering, and keyword and citation literature bursts. For CiteSpace analysis, the time slicing was configured from January 1, 2014, to December 31, 2024, with a slice length of 1 year (years per slice = 1), and the selection criteria were set to extract the top 50 most frequently occurring items per slice (top N = 50) to construct the networks. Bibliometrix was used for visualization analysis of inter-country cooperation and the time evolution of author publication volume. In the country cooperation map, the thicker the line, the closer the cooperation between the two countries.

## Results

### Search results

**Figure 1:**
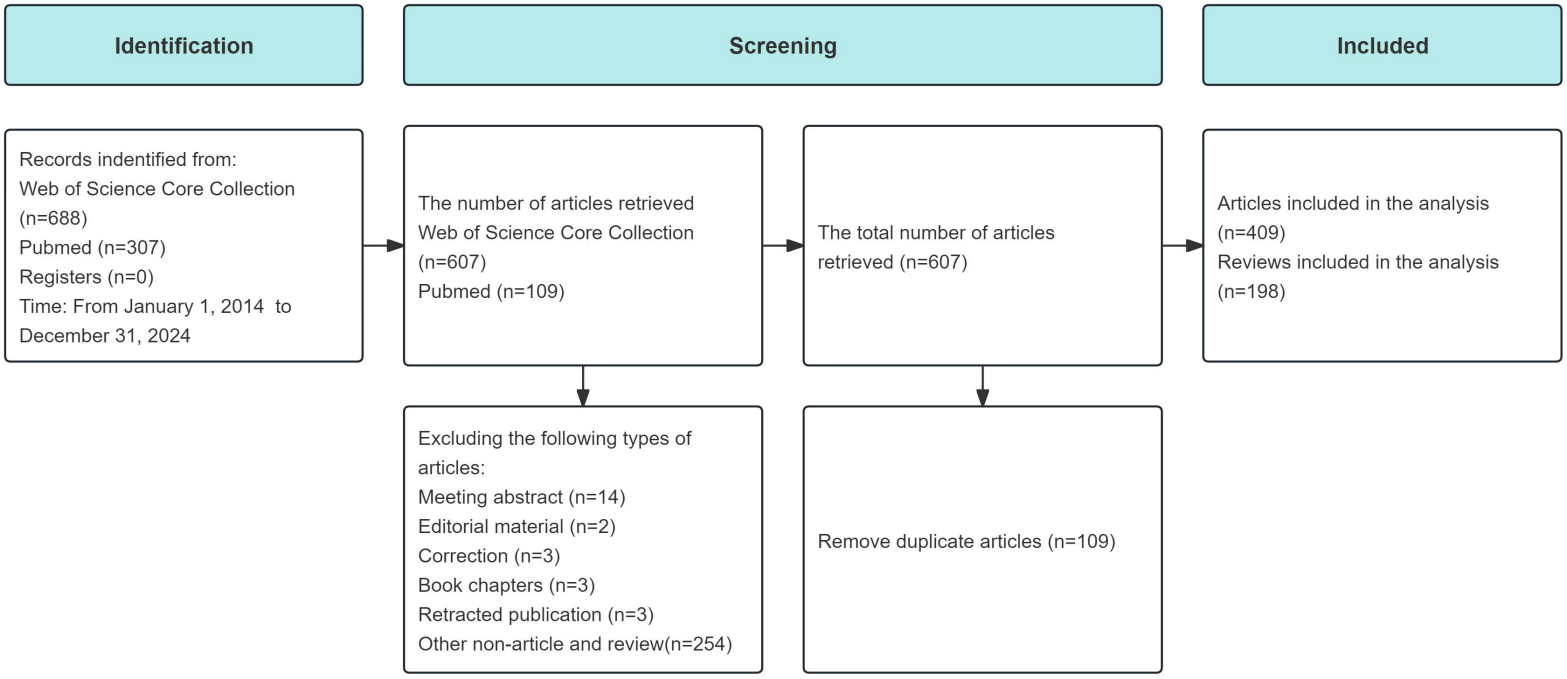
Flow diagram of the literature selection process. Records were identified from the Web of Science Core Collection and PubMed databases (2014 to 2024). After removing duplicates and excluding non-eligible document types, 607 publications were retrieved. Finally, 409 articles and 198 reviews were included in the analysis.

This study systematically searched the PubMed and WoSCC databases, identifying 607 research papers related to liver cancer and lipid metabolism. The literature search and screening process is illustrated in **Figure 1**. The selected papers were published between January 1, 2014, and December 31, 2024, encompassing contributions from 54 countries/regions and appearing in 304 academic journals. These papers involved 1,183 research institutions and 4,091 authors. The impact of this body of work is notable, with a cumulative citation count of 15,366 and an ACI of 25.31. These figures reflect broad international participation and a solid academic footprint in this field.

### Annual trend of publication volume

**Figure 2:**
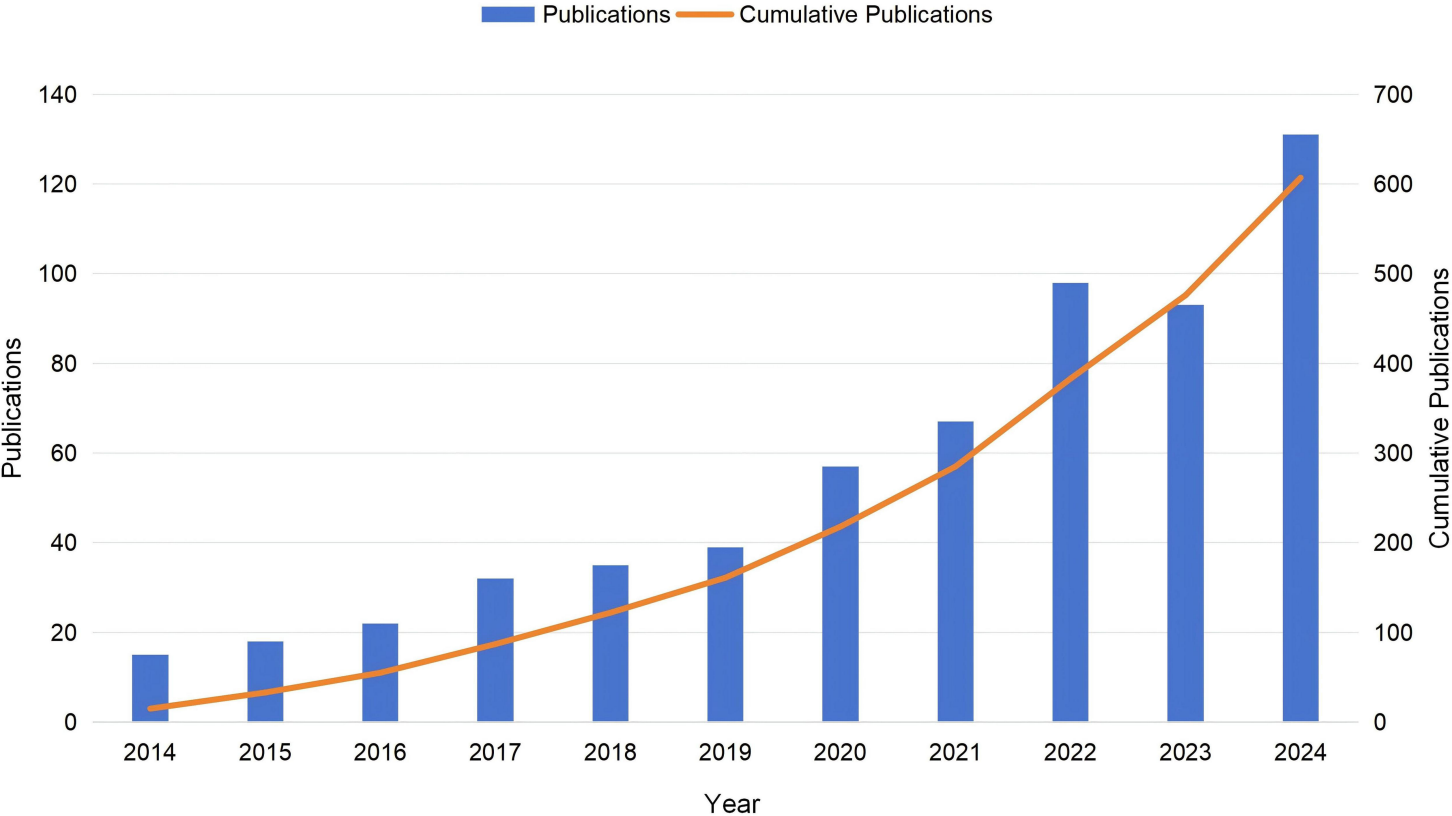
Annual and cumulative number of publications from 2014 to 2024. The blue bars represent the annual number of publications, corresponding to the left vertical axis. The orange line indicates the cumulative number of publications, corresponding to the right vertical axis.

The annual publication volume serves as a crucial quantitative indicator for evaluating the developmental trajectory of a research domain. As illustrated in **Figure 2**, literature from the past decade exhibits a significant upward trend in the number of publications concerning liver cancer and lipid metabolism. Notably, from 2014 to 2018, the publication output remained relatively low, totaling 122 articles, which constituted only 20% of the overall publications during this ten-year period. This pattern suggests that early investigations in this field were primarily exploratory. However, beginning in 2019, the publication volume experienced accelerated growth. This surge aligns with deeper investigations into tumor metabolic reprogramming, specifically the gradual elucidation of the molecular mechanisms linking lipid metabolism to the onset and progression of liver cancer. In 2024 alone, 131 articles were published, surpassing the cumulative total of the initial five-year period (2014–2018). In summary, this continuous upward trajectory reflects a growing and sustained academic focus on this intersection.

### Country analysis

To date, 54 countries/regions have contributed to research concerning lipid metabolism in liver cancer. **Table 1** details the top 10 most productive countries alongside their scientific influence indicators. In terms of publication volume, China (324 papers, 53.38%) and the United States of America (USA) (148 papers, 24.38%) exhibit the highest research output in this field. Regarding scientific influence, the USA maintains a substantial total citation count (6,004) and an ACI of 40.57, reflecting a strong academic impact. Germany also demonstrates a notable presence, with an ACI of 42.93 and a betweenness centrality of 0.25, suggesting its crucial role within the collaboration network. Furthermore, despite Japan’s relatively modest publication count (18 papers, 2.97%), its ACI reaches 45.22, indicating high per-paper quality and influence. Overall, an analysis of the ACI metric reveals that research outputs from developed countries frequently exhibit higher average citation rates.

**Table 1:** Top 10 productive countries.

Rank	Country	Quantity	Proportion%	Citations	ACI	H-index	Centrality
1	China	324	53.38	6,710	20.71	45	0.15
2	USA	148	24.38	6,004	40.57	44	0.18
3	Germany	46	7.58	1,975	42.93	22	0.25
4	Italy	29	4.78	1,111	38.31	16	0.09
5	France	24	3.95	944	39.33	15	0.13
6	India	22	3.62	356	16.18	10	0.08
7	South Korea	22	3.62	643	29.23	11	0.02
8	UK	21	3.46	718	34.19	13	0.14
9	Japan	18	2.97	814	45.22	10	0.02
10	Canada	15	2.47	424	28.27	11	0.10

ACI, average citations per item; USA, United States of America; UK, United Kingdom. Centrality refers to betweenness centrality, an indicator measuring the importance of a node as a bridge in the collaboration network (nodes with betweenness centrality ≥ 0.1 are generally considered key hubs).

**Figure 3:**
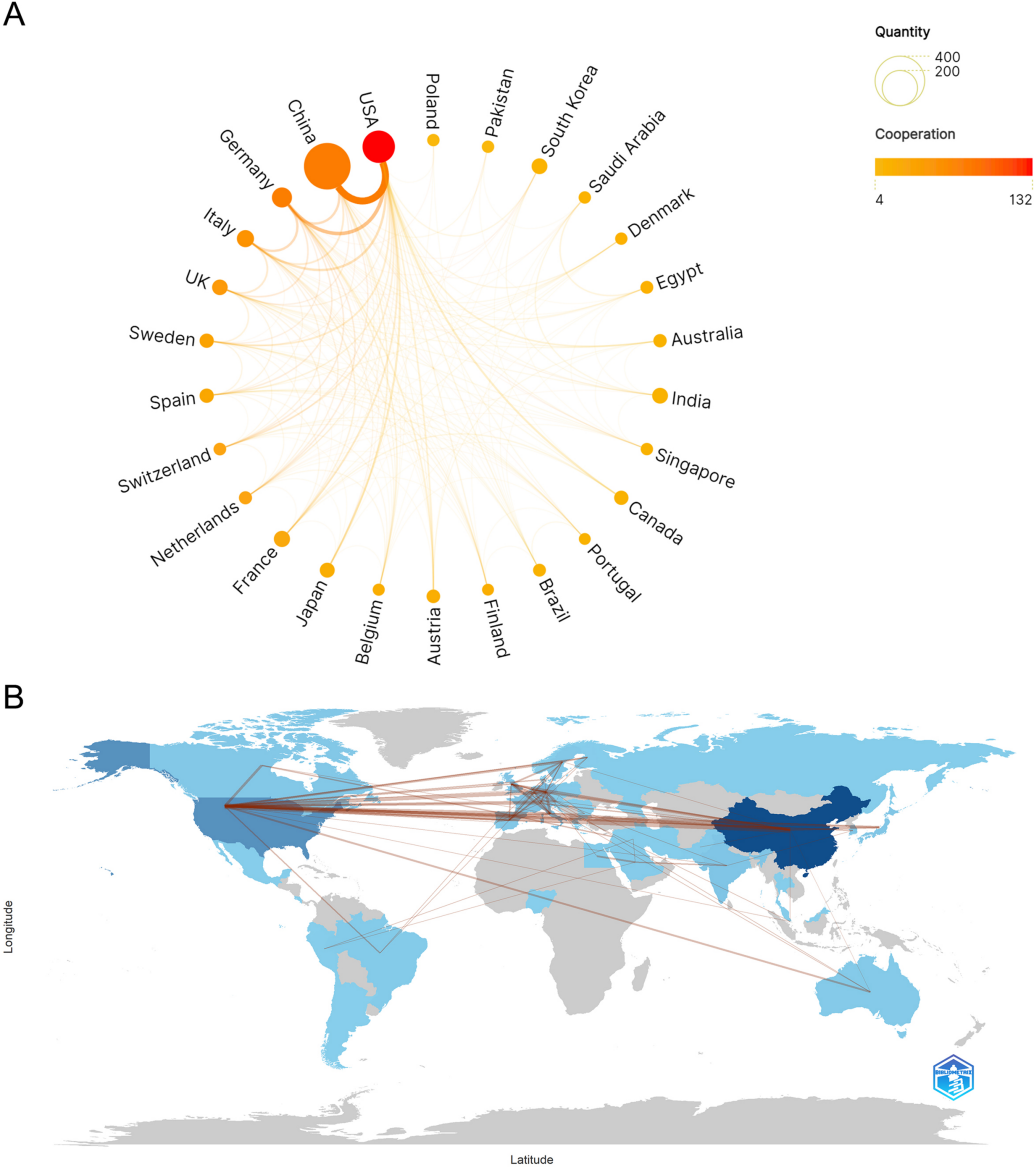
Analysis of country and regional collaboration networks. **(A)** Scimago Graphica collaboration visualization map of countries/regions. A larger node suggests that the country/region has a higher publication output. A node color closer to red indicates a higher degree of international collaboration, and a thicker connecting line suggests a closer collaborative relationship between the two countries/regions. **(B)** Country/region collaboration map. The darker the blue color, the greater the publication volume of the country. The thickness of the connecting line represents the strength of the scientific cooperation, with a thicker line indicating a stronger partnership.

Visual analyses of the scientific cooperation networks among various countries/regions were conducted using Scimago Graphica and the Bibliometrix software package. As illustrated in **Figure 3A**, the size of each node corresponds to the total publication output of the respective country, whereas the color gradient, transitioning from yellow to red, indicates the total link strength and reflects the extent of international collaboration. Notably, although China presents the largest node indicative of the highest publication volume, the USA exhibits the darkest red node, suggesting the greatest degree of international collaborative engagement. In the global collaboration map (**Figure 3B**), the blue color intensity of each country is proportional to its publication volume, and the thickness of the connecting lines visually represents the strength of scientific cooperation between nations. The analysis reveals that China and the USA share the thickest connection line, thereby establishing the most prominent collaborative relationship and forming the core of the global research network. Furthermore, robust scientific cooperation is also evident between the USA and European nations, particularly Germany.

### Institutional analysis

This study conducted a systematic analysis of research institutions in the global field of liver cancer and lipid metabolism. As shown in **Table 2**, we selected the top 10 most representative research institutions in this domain based on the number of published papers. In terms of national distribution, Chinese institutions dominated, with a total of 6 institutions included. Among these, the Chinese Academy of Sciences ranked second with 19 published papers. American institutions also performed well, with the University of California system and the National Institutes of Health (NIH) ranking first and seventh respectively, with 20 and 15 published papers.

**Table 2:** Top 10 productive institutions.

Rank	Institution	Country	Quantity	Citations	ACI	H-index
1	University of California System	USA	20	811	40.55	13
2	Chinese Academy of Sciences	China	19	902	47.47	13
3	National Institute of Health and Medical Research	France	18	836	46.44	13
4	Fudan University	China	16	530	33.13	11
5	Zhejiang University	China	16	410	25.63	11
6	Huazhong University of Science and Technology	China	15	293	19.53	8
7	National Institutes of Health	USA	15	1,625	108.33	12
8	Shanghai Jiao Tong University	China	13	553	42.54	10
9	Capital Medical University	China	11	102	9.27	5
10	National Cancer Institute	USA	11	1,540	140.00	9

ACI, average citations per item; USA, United States of America.

In terms of research influence, significant differences were observed among various institutions. The National Cancer Institute (NCI) demonstrated outstanding academic influence, boasting the ACI at 140.00, followed closely by the NIH with an ACI of 108.33. Both institutions far exceeded the others in this metric. The Chinese Academy of Sciences and INSERM in France also ranked highly, with ACI of 47.47 and 46.44, respectively. Notably, although Chinese institutions maintained a distinct advantage in publication volume, the ACI for several of these institutions (such as Zhejiang University, Huazhong University of Science Technology, and Capital Medical University) remained relatively low. This indicates that there is still room for improvement regarding the academic impact of their research outputs. Finally, the H-index analysis revealed that the University of California System (13), the Chinese Academy of Sciences (13), and INSERM in France (13) demonstrated the highest sustained academic influence in this field.

**Figure 4:**
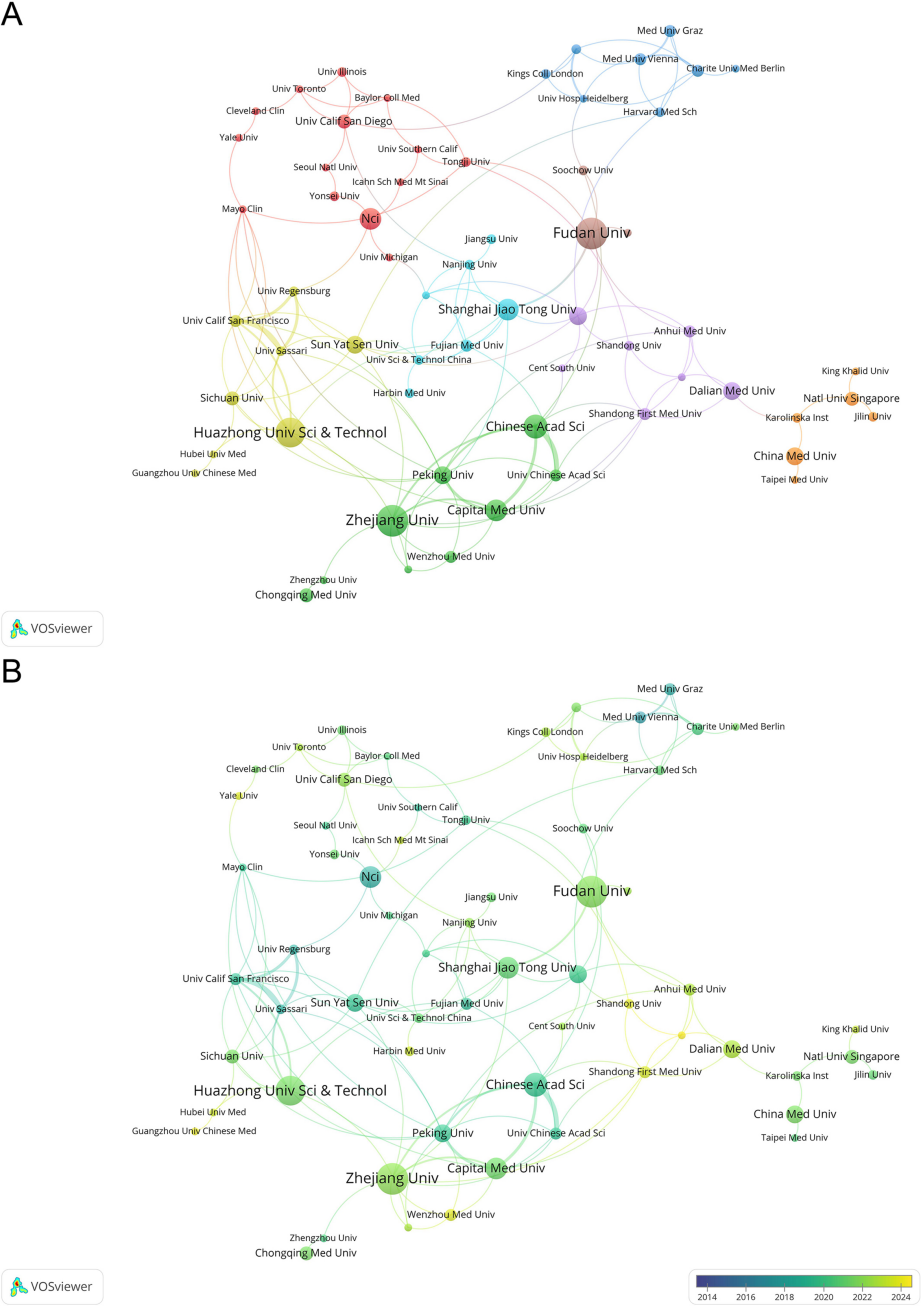
Institutional collaboration network generated by VOSviewer. **(A)** Network visualization map. Each node represented an institution, and the size of the node reflected its publication output. Different colors indicate distinct collaboration clusters. The thickness of the connecting lines denotes the strength of collaboration between institutions. To ensure visual clarity, the minimum number of occurrences for institutional nodes was set to 4. **(B)** Overlay visualization map of the institutional collaboration network. The color gradient of the nodes, ranging from blue to yellow, represents the average publication year (2014 to 2024), illustrating the temporal evolution of institutional research activity.

Through the analysis of the institutional collaboration network (**Figure 4A**), a dense and well-connected research network was observed, with Fudan University serving as one of the central hubs. Zhejiang University, Chinese Academy of Sciences, Shanghai Jiao Tong University, and Huazhong University of Science and Technology also occupy prominent positions within the network, forming the core of domestic collaboration in China. In terms of international cooperation, the NCI acts as an important bridging node, linking multiple institutions in the USA, Europe, and Asia. Several European institutions, including Medical University of Vienna and Charité–Universitätsmedizin Berlin, form interconnected clusters while maintaining collaborative ties with leading Chinese institutions. The overlay visualization further indicates that recent research activity has been increasingly concentrated in Chinese institutions (**Figure 4B**), reflecting their growing influence in this field.

### Journal analysis

As illustrated in **Table 3**, a systematic analysis of the contributions of journals in the domain of liver cancer and lipid metabolism research reveals that the majority of research findings in this area are published in reputable journals specializing in oncology and molecular biology. *Cancers* and the *International Journal of Molecular Sciences* rank highest, with 20 and 18 publications, respectively, underscoring the significant prominence of these journals. Notably, although the *Journal of Hepatology* ranks sixth in terms of publication volume (10 articles), it boasts a remarkably high impact factor (IF) of 26.8, with an ACI of 61.40, reflecting the journal’s authority and influence in the field. Regarding journal quality, seven of the top ten journals are classified in the Q1 category of the journal citation reports, with *Cancer Research* distinguished by an ACI of 73.00 and an IF of 12.5, highlighting its exceptional academic influence.

**Table 3:** Top 10 journals.

Rank	Journal	Quantity	ACI	IF	JCR
1	Cancers	20	15.90	4.50	Q1
2	International Journal of Molecular Sciences	18	68.00	4.90	Q1
3	Frontiers in Pharmacology	13	20.92	4.40	Q1
4	Cells	11	28.36	5.10	Q2
5	Frontiers in Oncology	11	18.45	3.50	Q2
6	Journal of Hepatology	10	61.40	26.80	Q1
7	Oncology Letters	10	17.60	2.50	Q3
8	Scientific Reports	10	27.40	3.80	Q1
9	Hepatology	9	33.22	12.90	Q1
10	Cancer Research	7	73.00	12.50	Q1

ACI, average citations per item; IF, impact factor; JCR, journal citation reports.

**Figure 5:**
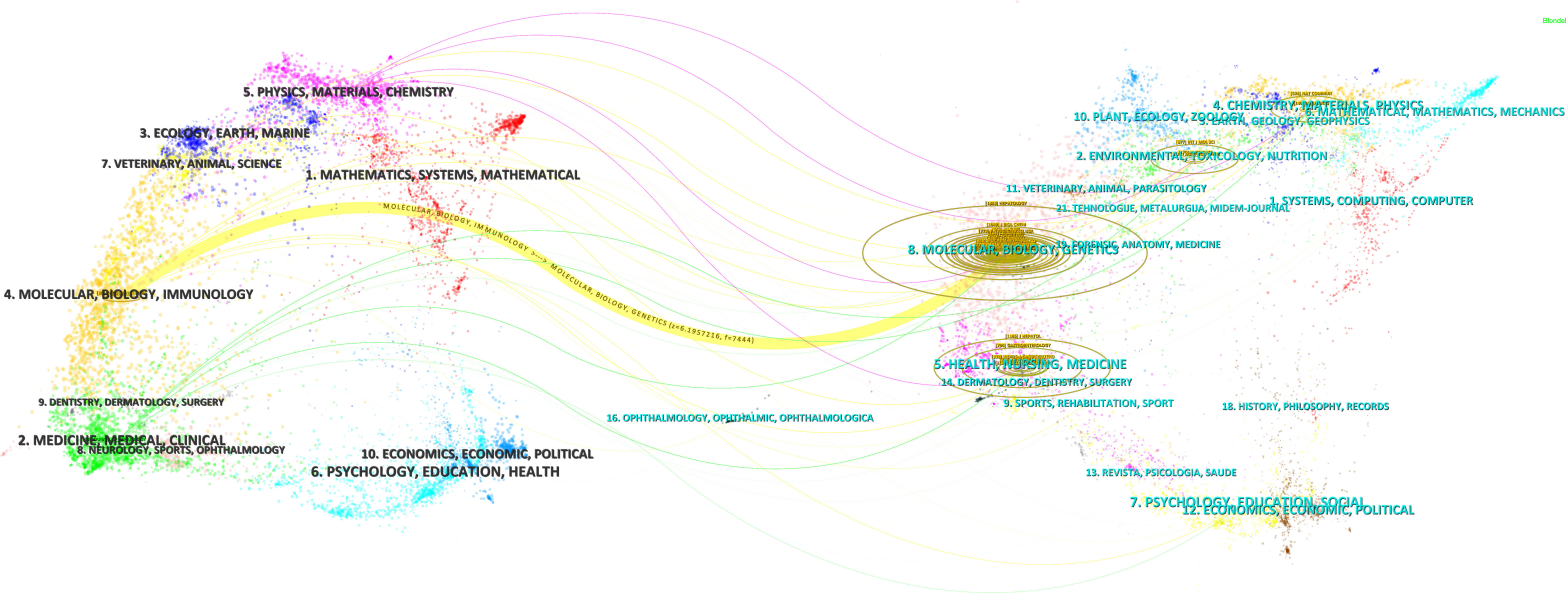
The dual-map overlay of journal publishing research. The length of the horizontal axis of the ellipse represents the number of authors, and the length of the vertical axis reflects the number of papers published by the journal. The colored curves indicate the knowledge flow and citation paths between different disciplinary journals.

To reveal the interdisciplinary characteristics of liver cancer and lipid metabolism research, we employed a dual-map overlay analysis method (**Figure 5**). This method constructs a two-layer network of citing journals (left) and cited journals (right) to visually illustrate the knowledge flow between disciplines. Nodes represent journals, while colored curves indicate the citation paths between different fields. The figure demonstrates that research findings from journals in molecular biology and immunology are frequently cited by studies published in molecular biology and genetics journals.

### Author analysis

**Figure 6:**
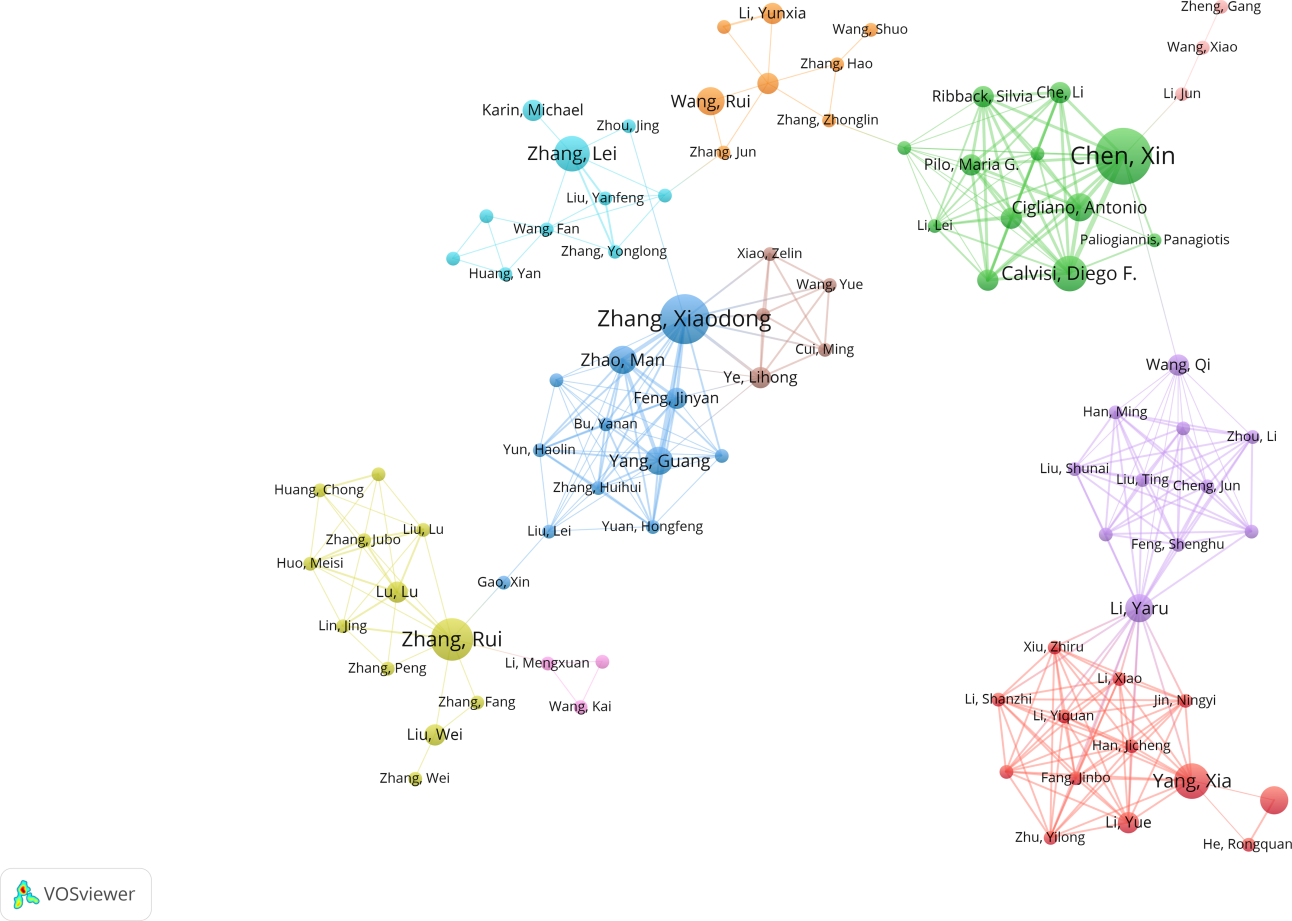
Analysis of author collaboration networks. The size of each node corresponds to the author’s publication output. Nodes sharing similar colors represent authors within the same collaborative cluster. The thickness of the connecting lines reflects the intensity of collaboration between pairs of authors. To ensure visual clarity and relevance, the minimum number of occurrences for authors was set to 2.

A total of 4,091 authors worldwide have contributed to publications concerning lipid metabolism and liver cancer. As presented in **Table 4** and **Figure 6**, the analysis of the most prolific authors reveals both their individual academic impacts and their collaborative networks. Zhang, Xiaodong, Francisco Nicolas Gonzalez, and Chen, Xin are the top three most productive authors, each contributing 5 publications, with an identical H-index of 5. Combining these metrics with the collaboration network highlights several distinct and closely-knit research groups. Specifically, the most prominent cluster is centered around Zhang, Xiaodong, who collaborates closely with other highly productive authors such as Zhao, Man and Feng, Jinyan. Similarly, an essential international collaborative team is formed by Chen, Xin and Cigliano, Antonio, both of whom rank among the top ten authors in publication volume. Furthermore, while some scholars may exhibit fewer network connections, their academic influence is exceptionally profound. Notably, Trauner, Michael and Francisco Nicolas Gonzalez boast remarkably high ACI of 166.25 and 162.80, respectively, underscoring the high quality and significant impact of their research contributions in this field.

**Table 4:** Top 10 authors.

Rank	Author	Country	Institution	Quantity	Citations	ACI	H-index
1	Zhang, Xiaodong	China	Tianjin University	5	480	96.00	5
2	Francisco Nicolas Gonzalez	Spain	Universidad de Cordoba	5	814	162.80	5
3	Chen, Xin	USA	University of California San Francisco	5	283	56.60	5
4	Alannan, Malak	France	Universite de Bordeaux	4	105	26.25	4
5	Merched, Aksam J.	France	Universite de Bordeaux	4	105	26.25	4
6	Cigliano, Antonio	Italy	University of Sassari	4	185	46.25	4
7	Zhao, Man	China	Tianjin Medical University	4	142	35.50	4
8	Trauner, Michael	Austria	Medizinische Universität Wien	4	665	166.25	4
9	Feng, Jinyan	China	Guizhou University	4	142	35.50	4
10	Kopsida, Maria	Sweden	Uppsala University	3	32	10.67	3

ACI, average citations per item; USA, United States of America.

### Citation analysis

Through a quantitative analysis of 607 research papers related to liver cancer and lipid metabolism, encompassing a total of 15,366 cited works, the top ten high-impact papers were selected based on citation frequency (**Table 5**). Among these, the most influential study, titled “NAFLD causes selective CD4+ T lymphocyte loss and promotes hepatocarcinogenesis”, was cited 578 times. This article elucidates the molecular mechanism by which non-alcoholic fatty liver disease (NAFLD) fosters the development of HCC through the reprogramming of lipid metabolism. The study confirmed that the abnormal accumulation of linoleic acid within the NAFLD microenvironment can induce mitochondrial dysfunction and selective depletion of CD4+ T cells, thereby compromising the body’s anti-tumor immune surveillance. This significant finding highlights the crucial interplay between metabolism and immunity in the pathogenesis of NAFLD-related liver cancer: disorders in lipid metabolism, particularly the imbalance of polyunsaturated fatty acid metabolism, not only inflict direct damage to liver cells but also establish a tumor-promoting microenvironment by disrupting CD4+ T cell homeostasis. This research provides a vital theoretical foundation for developing immune regulation strategies targeting lipid metabolism to prevent and treat NAFLD-HCC.

**Table 5:** Top 10 highly cited literatures.

Rank	Title	Citations	Year	DOI
1	NAFLD causes selective CD4+ T lymphocyte loss and promotes hepatocarcinogenesis	578	2016	10.1038/nature16969
2	Bile acid receptors as targets for drug development	538	2014	10.1038/nrgastro.2013.151
3	PPARs as metabolic regulators in the liver: Lessons from liver-specific PPAR-null mice	366	2020	10.3390/ijms21062061
4	Long noncoding RNA hulc modulates abnormal lipid metabolism in hepatoma cells through an mir-9-mediated RXRA signaling pathway	314	2015	10.1158/0008-5472.CAN-14-1192
5	HIF-1-mediated suppression of acyl-coa dehydrogenases and fatty acid oxidation is critical for cancer progression	278	2014	10.1016/j.celrep.2014.08.028
6	Deficient endoplasmic reticulum-mitochondrial phosphatidylserine transfer causes liver disease	254	2019	10.1016/j.cell.2019.04.010
7	The role of farnesoid X receptor in metabolic diseases, and gastrointestinal and liver cancer	253	2021	10.1038/s41575-020-00404-2
8	Ferroptosis in liver diseases: An overview	251	2020	10.3390/ijms21144908
9	miR-122 is a unique molecule with great potential in diagnosis, prognosis of liver disease, and therapy both as miRNA mimic and antimir	190	2015	10.2174/1566523214666141224095610
10	Lipid alterations in chronic liver disease and liver cancer	178	2022	10.1016/j.jhepr.2022.100479

DOI, digital object identifier.

By analyzing the co-cited papers (**Table 6**), we identified that the top ten papers predominantly focused on three research directions: cancer epidemiology studies, such as global cancer statistics; investigations into cancer characteristics, including reviews of cancer markers; and research on the molecular mechanisms linking lipid metabolism and cancer. Notably, the literature on global cancer statistics, which was co-cited 61 times, served as an authoritative reference for liver cancer epidemiology research. In contrast, the research on cancer markers, co-cited 39 times, established a theoretical framework for understanding the molecular characteristics associated with liver cancer development.

**Table 6:** Top 10 co-cited references.

Rank	Title	Citations	Year	DOI
1	Global cancer statistics 2020: Globocan estimates of incidence and mortality worldwide for 36 cancers in 185 countries	61	2021	10.3322/caac.21660
2	Hallmarks of cancer: the next generation	39	2011	10.1016/j.cell.2011.02.013
3	Erratum: Global cancer statistics 2018: Globocan estimates of incidence and mortality worldwide for 36 cancers in 185 countries	37	2020	10.3322/caac.21609
4	Fatty acid synthase and the lipogenic phenotype in cancer pathogenesis	33	2007	10.1038/nrc2222
5	Increased lipogenesis, induced by AKT-mTORC1-RPS6 signaling, promotes development of human hepatocellular carcinoma	32	2011	10.1053/j.gastro.2010.12.006
6	Ferroptosis: an iron-dependent form of nonapoptotic cell death	30	2012	10.1016/j.cell.2012.03.042
7	Hepatocellular carcinoma	30	2021	10.1038/s41572-020-00240-3
8	Mechanisms of NAFLD development and therapeutic strategies	28	2018	10.1038/s41591-018-0104-9.
9	Lipid metabolism in cancer	27	2012	10.1111/j.1742-4658.2012.08644.x
10	Cellular fatty acid metabolism and cancer	25	2013	10.1016/j.cmet.2013.05.017

DOI, digital object identifier.

**Figure 7:**
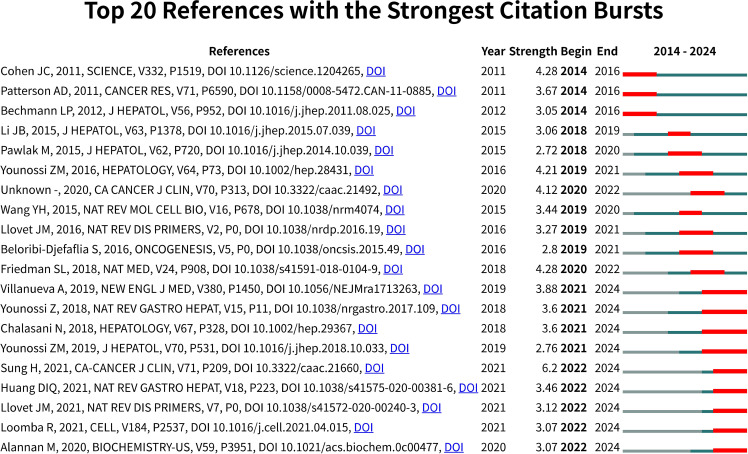
Top 20 references with the strongest citation bursts. The blue bar indicates the time interval, and the red bar indicates the duration of the citation burst period. The strength of the burst demonstrates the scientific impact and significance of the study within the field.

We employed the burst detection method to analyze the temporal distribution of citations, aiming to identify frequently cited papers within specific periods, as illustrated in **Figure 7**. Through the examination of 20 high-burst papers, we systematically delineated the developmental trajectory of liver cancer lipid metabolism research. During the foundational research phase from 2014 to 2016, Cohen et al. (26) and Bechmann et al. (27) clarified the aberrant mechanisms and pathological processes of lipid metabolism in liver cancer. In the clinical translation phase from 2018 to 2021, researchers such as Friedman et al. (28) and Younossi et al. (29) achieved significant breakthroughs in diagnostic criteria, treatment strategies, and molecular typing. Finally, during the multidisciplinary integration phase from 2021 to 2024, Loomba et al. (30), Llovet et al. (31), and Huang et al. (32) conducted comprehensive explorations of regulatory networks, biomarkers, and signaling mechanisms. The epidemiological study by Sung et al. (33), with a prominent burst intensity of 6.2, underscored the significance of this field and thoroughly illustrated the research transformation pathway from basic to clinical applications.

### Keyword analysis

This study utilized VOSviewer to analyze keywords that appeared more than five times in the dataset (**Table 7**). Among the top ten keywords, hepatocellular carcinoma, lipid metabolism, liver cancer, cancer, and metabolism were the most frequent, representing common terms in the field of liver cancer and lipid metabolism. Furthermore, the keywords “expression”, “nonalcoholic fatty liver disease”, “oxidative stress”, “activation”, and “nonalcoholic steatohepatitis” also demonstrated high frequencies, indicating their prominence in the research field concerning liver cancer and lipid metabolism.

**Table 7:** Top 10 Keywords.

Rank	Keywords	Quantity	Total link strength
1	hepatocellular carcinoma	251	1,134
2	lipid metabolism	162	748
3	liver cancer	133	633
4	metabolism	112	495
5	expression	109	519
6	cancer	100	437
7	nonalcoholic fatty liver disease	69	380
8	oxidative stress	64	285
9	activation	62	326
10	nonalcoholic steatohepatitis	62	336

**Figure 8:**
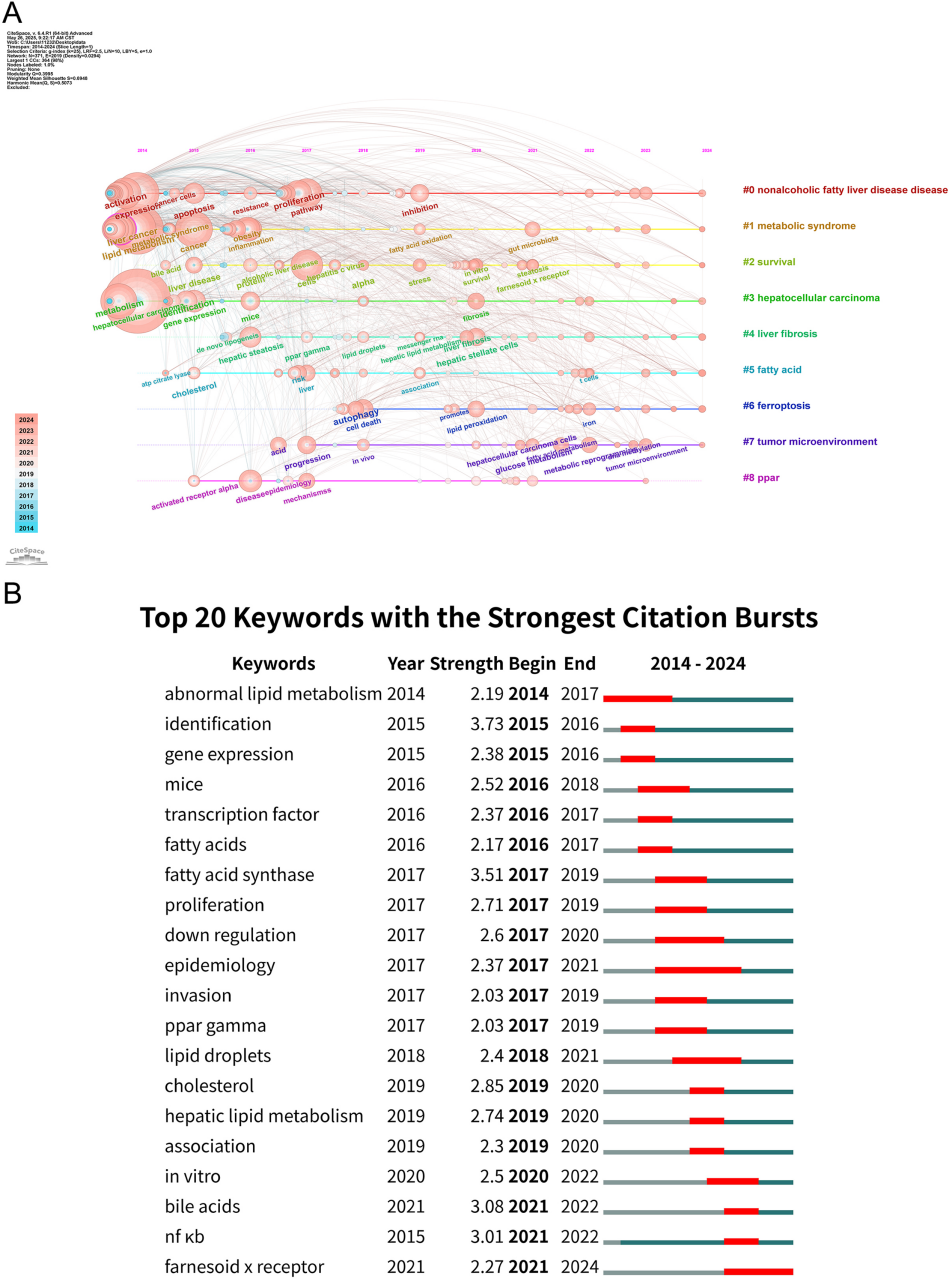
Analysis of keyword clusters and citation bursts. **(A)** CiteSpace visualization timeline view of keyword clustering analysis. The different colored horizontal lines represent distinct clusters formed by the keywords. The position of the nodes on the horizontal lines indicates the year in which the keyword first appeared, and the size of the node reflects its frequency. **(B)** Top 20 keywords with the strongest citation bursts. The blue bar indicates the time span of the keyword’s appearance, and the red bar indicates the duration of its burst period, reflecting the temporal evolution of research hotspots.

Additionally, this study employed CiteSpace to conduct a more in-depth analysis of the keywords present in related literature. Through keyword clustering, nine distinct clusters were generated (**Figure 8A**): cluster #0 “nonalcoholic fatty liver disease”, cluster #1 “metabolic syndrome”, cluster #2 “survival”, cluster #3 “hepatocellular carcinoma”, cluster #4 “liver fibrosis”, cluster #5 “fatty acid”, cluster #6 “ferroptosis”, cluster #7 “tumor microenvironment”, and cluster #8 “PPAR”. These clusters represent the current research hotspots and emerging trends.

Furthermore, twenty keywords with the strongest burst strength over the past decade were identified, as illustrated in **Figure 8B**. Among these, “differentiation” exhibited the highest burst strength with an index of 3.73, followed closely by “fatty acid synthase” and “lipid droplet”. Notably, “abnormal lipid metabolism and epidemiology” was the keyword with the longest burst duration.

## Discussion

### General information

A bibliometric analysis of the WoSCC over the past decade reveals that a total of 607 documents, comprising 409 research articles and 198 reviews, have been published in the field of lipid metabolism in liver cancer. These documents span 304 journals, involve 54 countries, 1,184 institutions, and 4,091 authors. Notably, the number of publications in the past three years has reached 322, accounting for 53.05% of the total, which indicates a significant growth trend and underscores that this field has emerged as a prominent area of research in liver cancer. Geographical analysis indicated that China leads with 324 publications (53.38%); however, the USA has the highest ACI at 40.57, while Germany, with a betweenness centrality of 0.25, has established itself as a key academic hub. This significant discrepancy between high publication volume and lower citation impact among certain institutions requires critical synthesis. The dominance of Chinese institutions in publication volume predominantly reflects a massive output of foundational mechanistic and *in vitro* studies. Conversely, the sustained high citation impact of Western institutions is often driven by paradigm shifting, international multicenter clinical trials and authoritative epidemiological guidelines. This highlights a prominent qualitative gap in the current research landscape: the stagnation in translating robust basic mechanistic discoveries into clinical targeted therapies. Bridging this translational gap is the most critical challenge for future research.

### Hotspots and frontiers

To systematically reveal the research hotspots in the field of lipid metabolism in liver cancer, this study conducted a comprehensive review of the current research status through keyword frequency analysis, burst detection, and spatio-temporal clustering analysis, combined with the citation burst characteristics of highly cited literature. Notable research includes the 2016 publication in Nature titled “NAFLD causes selective CD4(+) T lymphocyte loss and promotes hepatocarcinogenesis”, which elucidated the mechanism by which abnormal lipid metabolism fosters liver cancer through the selective loss of CD4(+) T lymphocytes, providing crucial evidence for understanding the relationship between lipid metabolism and tumorigenesis (34). To ensure our interpretation is powerfully grounded in the bibliometric data rather than generic summaries, the following future research directions are directly linked to our quantitative findings (1): The mechanisms and therapeutic potential of PPARγ, which aligns with the specific emergence of cluster #8 PPAR in this study; (2) The molecular pathways of FXR, directly supported by the high burst strength of the keywords “bile acids” and “farnesoid X receptor” identified in this study; (3) The molecular mechanisms by which the NF-κB signaling pathway mediates lipid metabolism reprogramming.

### Oxidative stress and nonalcoholic fatty liver disease

NAFLD encompasses a spectrum of liver disorders characterized by abnormal lipid accumulation in hepatocytes. This condition ranges from simple steatosis to nonalcoholic steatohepatitis (NASH), the latter of which is marked not only by fat accumulation in hepatocytes but also by accompanying liver inflammation and damage (35, 36). The global prevalence of HCC associated with NAFLD is expected to rise in parallel with the increasing prevalence of obesity (32). Oxidative stress (OS) plays a pivotal role in the progression of NAFLD, defined as the excessive accumulation of reactive oxygen species (ROS) that surpasses the body’s antioxidant clearance capacity, leading to cellular damage (37). In chronic liver diseases, persistent oxidative stress not only promotes carcinogenesis through direct damage to DNA, lipids, and proteins but also facilitates the malignant transformation of hepatocytes by modulating the expression of oncogenes (38).

NAFLD-related HCC exhibits distinct metabolic-driven characteristics, with lipotoxicity and DNA oxidative damage induced by steatosis being particularly significant (39, 40). The development of liver steatosis is closely associated with endoplasmic reticulum (ER) stress, which can directly promote the generation of reactive oxygen species (ROS) and induce oxidative stress, thereby exacerbating inflammatory responses and genomic instability (41). Throughout the progression of NAFLD, the excessive accumulation of free fatty acids serves as a critical factor leading to lipotoxicity and mitochondrial dysfunction. When ROS accumulation surpasses the physiological threshold, it can initiate a liver inflammatory cascade through multiple mechanisms: damaged hepatocytes release pro-inflammatory cytokines (such as TNF-α and IL-6) to activate Kupffer cells, while circulating monocytes and neutrophils are recruited to the liver, further releasing inflammatory mediators, nitric oxide (NO), and ROS, which amplify the inflammatory response (42). Furthermore, lipid metabolism disorders and oxidative damage in NAFLD can induce a selective reduction of CD4(+) T lymphocytes, thereby weakening immune surveillance and increasing the risk of HCC (34). In conclusion, liver steatosis, oxidative stress, chronic inflammation, and immune dysregulation mutually reinforce each other, creating a vicious cycle that collectively drives the progression of NAFLD to NASH and HCC.

### PPARγ

PPARγ, a member of the peroxisome proliferator-activated receptor family, plays a crucial role in adipocyte differentiation, lipid storage, and glucose homeostasis by regulating the transcription of genes associated with these processes (43, 44). Recent loss-of-function experiments concerning fat synthesis have established that PPARγ is essential for promoting adipocyte differentiation and facilitating lipid accumulation in adipocytes (45). Furthermore, studies indicate that the knockout of PPARγ results in lipid metabolism disorders, characterized by elevated triglyceride and total cholesterol levels, alongside decreased high-density lipoprotein cholesterol (46). Research has confirmed that ZBTB20 interacts with PPARγ, activating the WNT/CTNNB1 signaling pathway and its downstream effectors, which are implicated in HCC tumorigenesis (47). Additionally, recent findings (48) highlight PPARγ’s role as a key transcription factor within the tumor microenvironment, where it drives the terminal differentiation of macrophages into MMP9+ tumor-associated macrophages (TAMs). Stabilization of PPARγ has been shown to inhibit the progression of HCC (49, 50). However, comparing these identified bibliometric hotspots to actual therapeutic developments reveals a significant clinical stagnation. Clinical therapies targeting PPARγ remain largely unrealized due to the expression heterogeneity of PPARγ across different liver cancer subtypes and the lack of tumor specific targeted delivery methods. Systemic administration of agonists risks exacerbating overall metabolic dysregulation, necessitating further investigation to overcome these specific translational hurdles.

### FXR

One of the fundamental metabolic functions of the liver is the production of bile acids. Up to 40% of patients with HCC present with cholestatic jaundice, indicating a close relationship between bile acid metabolism disorders and the occurrence of liver tumors (51). The FXR, a bile acid-sensing nuclear receptor, serves as a major transcriptional regulator of bile acid synthesis and excretion (52). As a central regulator of bile acid homeostasis, FXR modulates the production and excretion of bile acids to prevent cholestasis. While FXR emerges as a breakthrough theoretical target in HCC research, its clinical translation has been conspicuously delayed. The fundamental reason FXR research has not yet yielded effective therapies lies in the complex clinical realities of systemic activation. Activating FXR can cause severe clinical side effects, including pruritus and deleterious shifts in lipid profiles (53). Furthermore, existing studies have demonstrated that the abnormal activation of the transcriptional coactivator YAP within the tumor microenvironment can induce inhibitory epigenetic reprogramming of the FXR gene (54, 55). This complex compensatory mechanism partly explains why relying solely on FXR-targeted therapies has been challenging in clinical settings. Therefore, developing liver targeted drug delivery systems and combinatorial therapies addressing YAP dependent resistance represent the necessary next steps for clinical translation.

### NF-κB

NF-κB is primarily localized in adipose tissue and serves as a crucial molecule that connects inflammatory responses with tumorigenesis. It plays a pivotal role in regulating the progression of liver diseases, including liver injury, liver fibrosis, and HCC, and is essential for the development of liver cancer (56). When NF-κB is excessively activated, it leads to the accumulation of lipid peroxidation products, promotes the release of numerous inflammatory factors, thereby exacerbating the inflammatory response in the liver (57). However, NF-κB exhibits a significant “double-edged sword” effect in the pathological and physiological processes of the liver (58). In cases of acute liver injury, moderate activation of NF-κB can protect liver cells by upregulating the expression of antioxidant genes and anti-apoptotic proteins. Conversely, in chronic liver diseases, its persistent activation stimulates the release of inflammatory factors and promotes cell proliferation, thereby accelerating the progression towards fibrosis and liver cancer (59). This dual role is closely associated with the degree of activation, duration of action, and the surrounding microenvironment. Although therapeutic strategies targeting NF-κB have demonstrated anti-inflammatory potential in animal models, comprehensive clinical inhibition may severely compromise the body’s immune defense functions (60, 61). Therefore, future research must concentrate on developing selective regulatory methods to achieve precise interventions, moving beyond broad basic inhibition toward context specific clinical applications.

### Limitations and prospects

This study is the first to employ bibliometric methods to systematically analyze the current research status in the field of liver cancer and lipid metabolism. However, it has several limitations. Firstly, the data source is restricted to the WoSCC database, which may not encompass all relevant literature in this field. Secondly, the literature screening criteria are confined to English publications, potentially introducing language bias. Thirdly, due to the continuous updates of the Web of Science database, metrics such as citation frequency and H-index of articles are in a dynamic state of flux, which may impact the timeliness of the research findings. Despite these limitations, this study, through a systematic analysis of existing literature, provides a significant reference for the in-depth exploration of the molecular mechanisms underlying liver cancer and lipid metabolism, and offers valuable insights for future research directions.

## Conclusion

This study systematically elucidates the evolving trends in liver cancer and lipid metabolism research from 2014 to 2024. Bibliometric indicators and keyword clusters reveal that current research predominantly focuses on the molecular mechanisms of oxidative stress in nonalcoholic fatty liver disease. While citation bursts highlight targets such as the FXR and PPARγ as major frontiers, a significant gap remains between these foundational mechanistic discoveries and clinical applications. Future research must prioritize overcoming these clinical translational barriers by developing targeted delivery systems and precise combinatorial therapies.

## Data Availability

The raw data supporting the conclusions of this article will be made available by the authors, without undue reservation.
